# Fractured Spinal Needle During Cesarean Section: A Case Report

**DOI:** 10.7759/cureus.96826

**Published:** 2025-11-14

**Authors:** Paulo Correia, Teresa Pereira, Carla Fernandes

**Affiliations:** 1 Anesthesiology, Unidade Local de Saúde de Entre o Douro e Vouga, Santa Maria da Feira, PRT

**Keywords:** cesarian section, needle fracture, obstetric anesthesia, scoliosis, spinal anesthesia

## Abstract

Spinal needle fracture during neuraxial anesthesia is a rare but potentially serious complication. We report the case of a term primigravida with scoliosis and obesity who underwent labor epidural analgesia followed by spinal anesthesia for cesarean section. Multiple attempts were required before cerebrospinal fluid return was achieved, and a local anesthetic was administered. Upon withdrawal, the spinal needle fractured, leaving a fragment in the paravertebral musculature. The fragment was successfully removed endoscopically on postpartum day three without sequelae. This case highlights risk factors, the importance of imaging, appropriate management, and preventive strategies, including the potential role of neuraxial ultrasound.

## Introduction

Neuraxial anesthesia is the preferred technique for obstetric procedures due to its safety profile and ability to maintain maternal consciousness and airway protection. Although overall complication rates are low, rare mechanical iatrogenic complications such as spinal needle fracture can occur and may have significant consequences if not promptly recognized and managed [1,2].

Risk factors include difficult anatomy, obesity, scoliosis, multiple attempts, and excessive manipulation or force during needle insertion or withdrawal [1-4]. Reporting such cases contributes to improved awareness and prevention strategies [1,5]. We present a case of spinal needle fracture during spinal anesthesia for cesarean delivery, and discuss contributing factors, diagnostic evaluation, management, and prevention.

## Case presentation

A 40-week and one-day primigravida in active labor with spontaneous rupture of membranes was admitted for delivery. Her medical history was significant for scoliosis and obesity (BMI 34.3 kg/m²) but otherwise unremarkable. Attempts were made to obtain X-ray imaging for characterization of the scoliosis, but no images were available at the time of admission.

Epidural analgesia was requested for labor. The first attempt resulted in blood aspiration, while the second attempt at the L3-L4 interspace using an 18G Tuohy needle and loss-of-resistance to saline was successful. Labor analgesia was initiated, but pain relief remained suboptimal over the following hours.

Due to failure of labor progression and intra-partum fever, cesarean section was indicated. After testing the epidural catheter and confirming inadequate block quality, the decision was made to remove it. The anesthesiologist elected to perform spinal anesthesia using a 27G Whitacre needle (B. Braun Pencan®, Bethlehem, PA, 88 mm) with an introducer at the same interspace. The procedure proved technically difficult, requiring three attempts before cerebrospinal fluid (CSF) was successfully obtained. Because the CSF reflux appeared slower than usual, the anesthesiologist elected to administer only an intrathecal analgesic dose of local anesthetic. However, upon attempted needle withdrawal, unexpected resistance was encountered, and the needle fractured. Only the proximal half was retrieved, leaving the distal 4-cm fragment within the paravertebral region.

General anesthesia was induced to proceed with the cesarean section, which was completed uneventfully. Postoperatively, the patient remained asymptomatic, with no back pain, headache, fever, sensory or motor deficits, or neurological abnormalities on physical examination.

**Figure 1:**
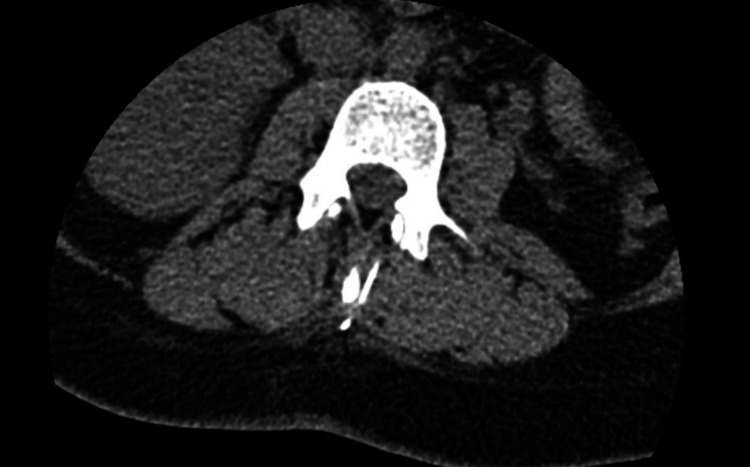
Axial CT scan at the L3 level showing the metallic fragment lodged in the paravertebral musculature, approximately 1 cm lateral to the spinous process.

A computed tomography (CT) scan was performed, which confirmed the presence of a linear metallic fragment consistent with the broken spinal needle located within the paravertebral musculature (Figure 1). There was no involvement of the spinal canal, no signs of hematoma, and no additional complications were identified.

On postpartum day 3, the patient underwent minimally invasive endoscopic surgery for fragment removal. The procedure was successful under general anesthesia, with no intraoperative or postoperative complications. At short-term follow-up (two weeks), the patient remained free of pain, neurological symptoms, or functional limitations.

Following this incident, the needle manufacturer was duly notified.

The patient provided written informed consent for publication of this case.

## Discussion

Spinal needle fracture is an exceptionally rare complication of neuraxial anesthesia, with only a limited number of cases reported in the literature [1,2]. However, when it occurs, it may pose diagnostic and therapeutic challenges. In this case, several well-established risk factors were present, including obesity, scoliosis, and multiple puncture attempts [1-4]. Anatomical variations and difficult landmark identification increase the likelihood of needle deflection, bending, and mechanical stress, potentially leading to fracture during insertion or withdrawal [1,2]. Similar reports describe that repeated attempts in technically challenging patients contribute significantly to this complication [2,4].

Management strategies depend on the location of the retained fragment, the presence of neurological symptoms, and the risk of migration. Imaging is crucial for accurate localization. CT is preferred due to its ability to precisely visualize metallic fragments and assess their relationship to the spinal canal [2,3]. In our case, CT confirmed the fragment was confined to the paravertebral musculature without canal involvement, allowing for planned surgical removal. Prior studies have shown that endoscopic or open surgical retrieval may be safely performed when the fragment is accessible and poses a potential risk if left in situ [3].

In asymptomatic patients with deep fragments or high surgical risk, conservative management may be considered, although this requires careful counseling and follow-up. In contrast, our patient was young, asymptomatic, and the fragment was superficially located, supporting surgical intervention. The favorable outcome observed in this case aligns with previous reports of successful retrieval without long-term sequelae [3].

Prevention remains paramount. Strategies include careful needle selection, minimizing the number of attempts, avoiding excessive force, and maintaining proper alignment of introducer and spinal needle [1-4]. Recent literature emphasizes the role of pre-procedural neuraxial ultrasound in patients with difficult anatomy [1,4,5]. Ultrasound can improve identification of the midline, estimate depth, and increase first-attempt success, reducing multiple punctures and mechanical complications [4,5]. Wider adoption of neuraxial ultrasound, especially in obese or scoliotic parturients, may decrease the risk of rare events such as needle fracture. Additionally, increased awareness, training, and adherence to best practices in neuraxial techniques can further enhance patient safety [1,5].

## Conclusions

Spinal needle fracture is a rare but clinically significant complication of neuraxial anesthesia. This case underscores the importance of recognizing patient-related risk factors such as obesity and scoliosis, as well as procedure-related factors such as multiple attempts. Prompt imaging and appropriate surgical management resulted in a favorable outcome without sequelae. Preventive strategies, including careful technique, appropriate needle handling, and consideration of neuraxial ultrasound in difficult anatomy, may reduce the risk of this uncommon but potentially serious event. Reporting such cases contributes to improved awareness, technique refinement, and safer anesthetic practice.
